# Characterisation of marsupial *PHLDA2 *reveals eutherian specific acquisition of imprinting

**DOI:** 10.1186/1471-2148-11-244

**Published:** 2011-08-19

**Authors:** Shunsuke Suzuki, Geoffrey Shaw, Tomoko Kaneko-Ishino, Fumitoshi Ishino, Marilyn B Renfree

**Affiliations:** 1 ARC Centre of Excellence for Kangaroo Genomics, The University of Melbourne, Parkville, Victoria 3010, Australia; 2 Department of Zoology, The University of Melbourne, Parkville, Victoria 3010, Australia; 3 Department of Epigenetics, Medical Research Institute, Tokyo Medical and Dental University, 1-5-45 Yushima, Bunkyo-ku, Tokyo 113-8510, Japan; 4 School of Health Sciences, Tokai University, Bohseidai, Isehara, Kanagawa 259-1193, Japan

## Abstract

**Background:**

Genomic imprinting causes parent-of-origin specific gene expression by differential epigenetic modifications between two parental genomes. We previously reported that there is no evidence of genomic imprinting of *CDKN1C *in the *KCNQ1 *domain in the placenta of an Australian marsupial, the tammar wallaby (*Macropus eugenii*) whereas tammar *IGF2 *and *H19*, located adjacent to the *KCNQ1 *domain in eutherian mammals, are imprinted. We have now identified and characterised the marsupial orthologue of *PHLDA2*, another gene in the *KCNQ1 *domain (also known as *IPL *or *TSSC3*) that is imprinted in eutherians. In mice, *Phlda2 *is a dose-sensitive negative regulator of placental growth, as *Cdkn1c *is for embryonic growth.

**Results:**

Tammar *PHLDA2 *is highly expressed in the yolk sac placenta compared to other fetal tissues, confirming a similar expression pattern to that of mouse *Phlda2*. However, tammar *PHLDA2 *is biallelically expressed in both the fetus and yolk sac placenta, so it is not imprinted. The lack of imprinting in tammar *PHLDA2 *suggests that the acquisition of genomic imprinting of the *KCNQ1 *domain in eutherian mammals, accompanied with gene dosage reduction, occurred after the split of the therian mammals into the marsupials and eutherians.

**Conclusions:**

Our results confirm the idea that acquisition of genomic imprinting in the *KCNQ1 *domain occurred specifically in the eutherian lineage after the divergence of marsupials, even though imprinting of the adjacent *IGF2-H19 *domain arose before the marsupial-eutherian split. These data are consistent with the hypothesis that genomic imprinting of the *KCNQ1 *domain may have contributed to the evolution of more complex placentation in the eutherian lineage by reduction of the gene dosage of negative regulators for both embryonic and placental growth.

## Background

Genomic imprinting produces monoallelic gene expression resulting from the parent-of-origin-dependent epigenetic modifications. Both DNA methylation and histone modifications are required to establish the paternal and maternal imprinting during development of the germ cells and to maintain it after fertilisation [1-4]. In humans and mice defects in some epigenetic modifiers or co-factors cause global disorders of genomic imprinting and of imprinted gene expression with early embryonic lethality, demonstrating that genomic imprinting is essential for mammalian development [5-10].

It is still unclear, however, why genomic imprinting has arisen in mammalian evolution, because adopting monoallelic gene expression means abandoning the merits of diploidy. In higher vertebrates, genomic imprinting has been found so far only in the viviparous therian mammals (eutherians and marsupials), but not in the egg-laying mammals, the monotremes [11,12]. Since only viviparous mammals have genomic imprinting, and many imprinted genes regulate fetal and placental growth, some authors have suggested that genomic imprinting is correlated with the evolution of mammalian viviparity [13-17]. It is therefore of great interest to compare genomic imprinting between eutherians and marsupials that diverged between 130 and 148 million years ago [18-20].

Most eutherians form a chorioallantoic (allantoic) placenta that is the site of highly efficient nutritional exchange between fetus and mother, allows lengthy intra-uterine growth, and in many cases supports the growth of a precocial young. In contrast, most marsupials depend on a relatively short-lived chorio-vitelline (yolk sac) placenta. Although often ignored, a yolk sac placenta is also present and functions for varying periods of time in all eutherian mammals [21-23]. Marsupials give birth to altricial young that are at a much earlier developmental stage than the neonates of most eutherians, but have developed a complex and advanced lactation system that supports further development and growth after birth, usually in a pouch [24,25].

Nearly 100 imprinted genes have been isolated in mice and humans. Imprinting has been studied in fourteen orthologues of these genes in marsupials but only 6 are imprinted [11,17,26-33]. These are *IGF2, IGF2R, PEG1/MEST, PEG10, INS *and *H19*, and are from 4 independent domains. We have previously reported that there is no evidence of genomic imprinting of *CDKN1C *(also known as *p57^KIP2^*) in a marsupial, the tammar wallaby (*Macropus eugenii*) [28,34]. *CDKN1C *is located in the *KCNQ1 *domain mapped adjacent to the *IGF2-H19 *domain in eutherians and marsupials. Genomic imprinting of the *IGF2-H19 *domain is highly conserved between eutherians and tammars [33]. Although the imprinting regulatory mechanisms of the *KCNQ1 *and *IGF2-H19 *domains are known to be independent in mouse, the two domains are only 300 kb distant from each other and both contain several important genes that control fetal and placental growth. Therefore, to confirm whether the only gene in the domain that is not imprinted in the tammar is the *CDKN1C *gene, we examined the imprinting status of the orthologue of the *PHLDA2 *gene from the tammar wallaby *KCNQ1 *domain.

*PHLDA2 *negatively controls growth of the chorioallantoic placenta in both human and mouse. In mice, deletion of *Phlda2 *causes placental overgrowth [35]. In contrast, biallelic expression of *Phlda2*, due to loss of imprinting, contributes to placental growth retardation and results in conceptuses with intrauterine growth restriction (IUGR) [36]. Furthermore, a single extra dose of *Phlda2 *has serious consequences for placental development, driving the loss of the junctional zone and reducing the amount of stored glycogen [37]. In humans, whilst there is silencing of *PHLDA2 *in complete hydatidiform moles [38], there is upregulation in placentae of fetuses with IUGR [39,40]; consistent with the results of genetic experiments in mice. Thus, the importance of gene dosage of *PHLDA2 *in eutherian placentation has been demonstrated by a number of studies. In this study, we characterise the orthologue of *PHLDA2 *in a marsupial, the tammar wallaby and examine its imprinting status in the chorio-vitelline placenta to clarify its possible contribution for the evolution of chorioallantoic placenta in the eutherian linage by dosage reduction consequent to acquisition of genomic imprinting.

## Results

### Characterisation of tammar *PHLDA2*

**Figure 1:**
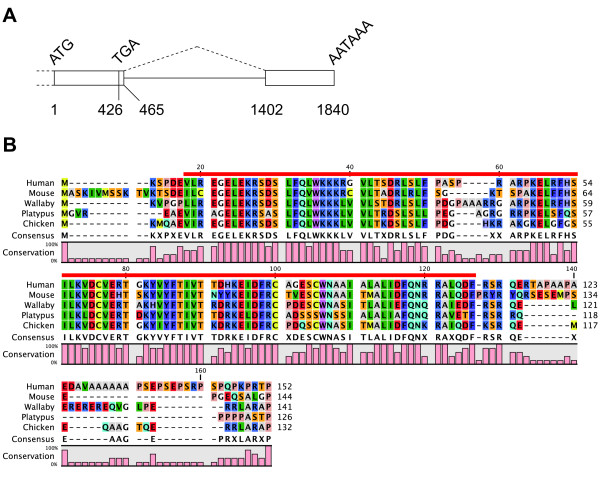
**Partial genomic structure and amino acid sequence of tammar PHLDA2**. (A) Exons are depicted as open boxes. The start and end of the ORF and the poly-A signal sequence are represented as ATG, TGA and AATAAA, respectively. The numbers indicate the nucleotide distance from the start of the ORF. (B) The alignment was created using "CLC Sequence Viewer 6" software. Identical and similar amino acids are indicated by the same color at each location. The bold red line represents the region of the PH domain. The amino acid sequences for human, mouse, tammar, platypus and chicken PHLDA2 were derived from the following accession numbers respectively: Genbank:NM_003311, Genbank:NM_009434, DDBJ:AB537423, GenBank:XM_001507454, GenBank:XM_421020.

A 272 bp fragment was amplified by RT-PCR using a primer pair designed to a highly conserved sequence in the open reading frame (ORF) of the *PHLDA2 *gene among multiple species. Given the PCR product sequence was highly similar to *PHLDA2 *of other species, we next carried out 3' RACE to obtain 3' UTR sequence of tammar *PHLDA2 *using the same forward primer used to amplify the 272 bp fragment as the gene specific primer. The 3' UTR (477 bp) of tammar *PHLDA2 *consisted of a short intron (937 bp) similar to eutherian *PHLDA2 *(Figure 1A). The expected genomic location of tammar *PHLDA2 *close to *CDKN1C *was confirmed by tammar BAC clone sequences in GenBank (NCBI). A 426 bp ORF encoding 142 amino acids was predicted with the supplemental sequence data from trace archive database (NCBI). Consistent with a previous comparison across vertebrates, that included fish, frog, chicken, mouse and human [41], the amino acid sequence of tammar PHLDA2 was also highly conserved within the PH (pleckstrin homology) domain, but there was lower conservation in the flanking sequences of both terminals (Figure 1B). The PH domain in tammar PHLDA2 shares 78% amino acid sequence similarity with human, 67% with mouse, 73% with platypus and 77% with chicken PHLDA2 orthologues.

### Tissue specific expression pattern of tammar *PHLDA2*

**Figure 2:**
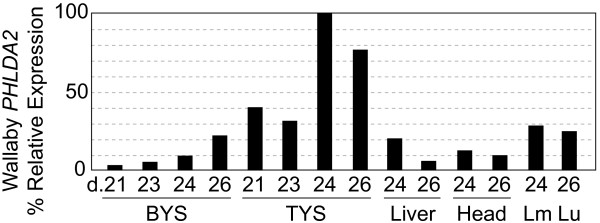
**Relative expression levels of tammar *PHLDA2 *mRNA**. The vertical axis represents the percentage expression levels when the highest result is regarded as 100%. The numbers on the horizontal axis indicate the day of gestation of each sample. "BYS" represents the bilaminar yolk sac; "TYS": trilaminar yolk sac; "Lm": limb and "Lu": lung.

As *PHLDA2 *is highly expressed in the yolk sac and placenta in human and mouse, we next analysed the expression pattern of tammar *PHLDA2 *in the yolk sac placenta as well as in several fetal tissues by quantitative PCR (QPCR). The marsupial yolk sac placenta consists of two regions, a bilaminar, avascular region and a trilaminar, vascular region. Both regions are the sites for fetal-maternal nutritional exchange while gases appear to be transferred principally via the vascular system of the trilaminar region [22,23,42,43]. The yolk sac placenta also synthesizes and stores nutrients required for fetal growth [22,23]. *PHLDA2 *mRNA expression in both bilaminar and trilaminar yolk sac was dramatically upregulated between day 24 to 26 of gestation (1-3 days before birth), although the relative expression level was lower in the bilaminar yolk sac (Figure 2). A lower level of tammar *PHLDA2 *expression was also observed in several fetal tissues, as observed for *Phlda2 *in the mouse, but not the human [44].

### Tammar PHLDA2 protein distribution in the yolk sac placenta

**Figure 3:**
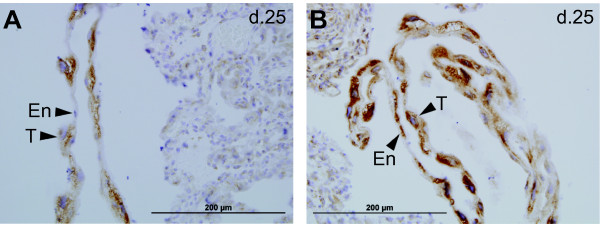
**PHLDA2 immunohistochemistry in the tammar yolk sac placenta**. Both bilaminar and trilaminar yolk sac membranes are shown as the cord like structure in the sections (A: bilaminar yolk sac, B: trilaminar yolk sac). "T" indicates trophoblast cells, the large cells with large nuclei, and "En" indicates yolk sac endodermal cells, the flat and thin cells with relatively small nuclei.

To confirm tammar wallaby PHLDA2 protein expression and distribution in the yolk sac placenta, we carried out immunohistochemistry using a mouse monoclonal antibody raised against a partial recombinant human PHLDA2. The immunogen included aa 1-110, encompassing the whole PH domain. There is a high degree of similarity of amino acid sequences between human and tammar PHLDA2 over this region (Figure 1B). Furthermore, we performed a genome-wide "TBLASTN" search for the published tammar genome sequence in the Ensembl database using the antigen peptide sequence for the query. It revealed the highest similarity of 76.5% for tammar PHLDA2 against whole sequence query (1-110/110 aa) as expected. The second highest hit was tammar PHLDA1, but this was aligned only partially (40-107/110 aa) with a much lower similarity of 51.5% for the aligned region. These data suggest that the immunostaining is positive for tammar PHLDA2 protein, although the possibility of some cross-reaction with PHLDA1 has not been completely excluded. Tammar PHLDA2 protein was present in both bilaminar and trilaminar regions in the yolk sac placenta, with strong immuno-staining in the cytoplasm of trophoblast cells of both parts of the yolk sac (Figure 3A, B), despite the substantially lower mRNA relative expression level in the bilaminar yolk sac (Figure 2).

### Allelic expression analysis of tammar *PHLDA2*

**Figure 4:**
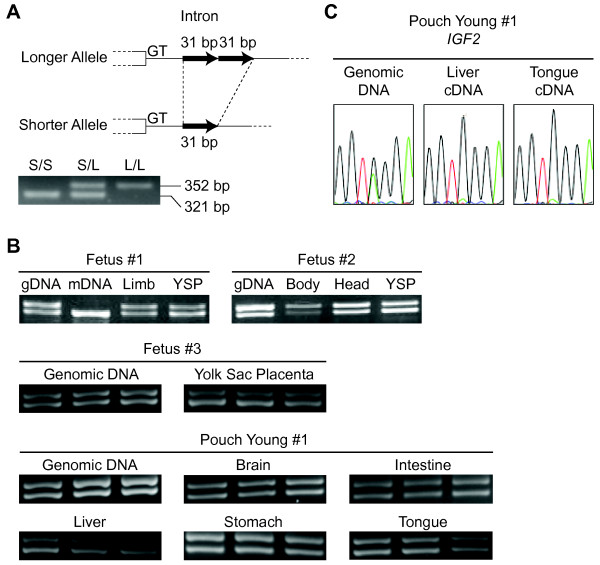
**Allelic expression analysis of tammar *PHLDA2***. (A) The difference of genomic structure by the length polymorphism is described. The open boxes represent exonic regions. Each black arrow represents a single 31 bp repeat unit. The longer allele has two units as a tandem repeat while the shorter allele has no repeat. The gel picture shows the PCR results using genomic DNA extracted from three different individuals having each genotype. "S" represents shorter allele; "L": longer allele. (B) Electrophoresis of RT-PCR products. Two different bands correspond to the amplified products from the longer and shorter allele. "gDNA" represents genomic DNA; "mDNA": maternal DNA; "YSP": yolk sac placenta. Three independent RT-PCR results were shown for the fetus #3 and pouch young #1. (C) The results of direct sequencing for tammar *IGF2 *PCR products amplified the unspliced transcript.

Finally, we analysed allelic expression pattern of tammar *PHLDA2 *to determine whether it was imprinted. We searched for polymorphisms to allow us to distinguish between the two parental alleles. No exonic polymorphisms were found in any of the individuals (n = 18) tested. However, there was a length polymorphism in some individuals in the intron characterised by the presence or absence of repeats in the 31 bp of intronic sequence (Figure 4A). Therefore, allelic expression could be determined directly by RT-PCR amplifying the unspliced *PHLDA2 *transcript using a primer pair designed to amplify the length polymorphic site. All RNA samples were DNase I treated and the lack of detectable contamination by genomic DNA was confirmed by PCR using the templates without reverse transcription (data not shown). Hence all intronic fragments amplified by RT-PCR were derived from unspliced transcripts, not from genomic DNA. The genomic PCR products showed that all four individuals were heterozygous for the length polymorphism and both alleles can be amplified equally (Figure 4B). All samples tested, had clear biallelic expression, demonstrating no evidence of genomic imprinting of tammar *PHLDA2 *(Figure 4B). On the other hand, monoallelic expression of tammar *IGF2 *could be confirmed by the amplification of the unspliced transcript using an intronic primer in the same way as the analysis on *PHLDA2 *(Figure 4C).

## Discussion

In this study, we identified and characterised the marsupial orthologue of *PHLDA2*. The amino acid sequence of tammar PHLDA2 shared highest conservation within the PH domain and lower conservation in the flanking sequences of both terminals, suggesting the essential role in the PH domain in contrast to the flanking regions, consistent with previous reports [41]. There was a similar high conservation of the amino acid sequences within the PH domain in the platypus, tammar and human PHLDA2, suggesting that this domain has a significant role with a similar function in marsupials and monotremes. The high level of mRNA expression in the trilaminar yolk sac and the protein localisation to the cytoplasm of trophoblast cells suggest that PHLDA2 functions in the tammar yolk sac placenta during pregnancy. However, although murine *Phlda2 *has the highest expression in the yolk sac [44], in mice with a disrupted *Phlda2 *gene the only abnormalities reported are in the chorioallantoic placenta [36]. Therefore, an ancestral role for PHLDA2 in the yolk sac might have been transferred to the chorioallantoic placenta during the evolution of the mouse.

There was no evidence of genomic imprinting of tammar *PHLDA2 *in this study. The mouse *Kcnq1 *domain forms a large imprinted gene cluster including *Phlda2, Slc22a18, Cdkn1c, Kcnq1, Ascl2 *(also known as *Mash2*) and some placenta-specific imprinted genes. However, we know now that at least two genes, *CDKN1C *and *PHLDA2 *that are involved in embryonic and placental growth in eutherians, are not imprinted in this marsupial [28]. Considering that both genes are located to the middle of the domain and that all the imprinted genes in this domain are co-ordinately regulated by a single imprinting centre in the mouse, our data strongly suggests that the whole *KCNQ1 *domain lacks genomic imprinting in marsupials. Interestingly, the *IGF2-H19 *imprinted domain, located adjacent to the *KCNQ1 *domain shares a highly conserved imprinting regulatory mechanism complete with a differentially methylated region and associated miRNA between eutherians and marsupials [33]. This study thus confirms that the origin of imprinting of the *KCNQ1 *domain evolved in the eutherian lineage after the divergence of marsupials, whereas that of the *IGF2-H19 *domain appeared before the marsupial-eutherian split, regardless of the close proximity of these two domains [34].

In the *Kcnq1 *domain of mice, while *Cdkn1c *is a negative regulator for embryonic growth [45], *Phlda2 *negatively controls placental growth [35-37] and acts as a true rheostat for placental growth [36]. Recently, using a single copy transgenic mouse, Tunster *et al*., (2010) reported that *Phlda2 *regulates extraembryonic energy stores. Two-fold over-expression of *Phlda2 *caused a 60% loss of the spongiotrophoblast layer with a 25-35% reduction of glycogen storage. Since acquisition of genomic imprinting of *PHLDA2 *in the *KCNQ1 *domain by silencing of the paternal allele was accompanied by gene dosage reduction in eutherians, this might have affected the evolution of placental structure and/or energy stores. In laboratory mice that have two active copies of *Phlda2 *with the second copy provided by the BAC transgene, there was only a slight progressive slowing of embryonic growth [37]. However, greater reduction of fetal growth may have been seen if the mice had had restricted food intake, as is often the case in the wild, so that limited nutrition would need to be partitioned between mother and fetuses. In this situation, reduced expression of *PHLDA2 *could have had a selective advantage through greater placental development. We hypothesise that acquisition of imprinting in the *KCNQ1 *domain in the ancestral line that gave rise to the eutherian mammals may have allowed increased the placental growth and extended gestation that characterises this group of mammals.

## Conclusions

**Figure 5:**
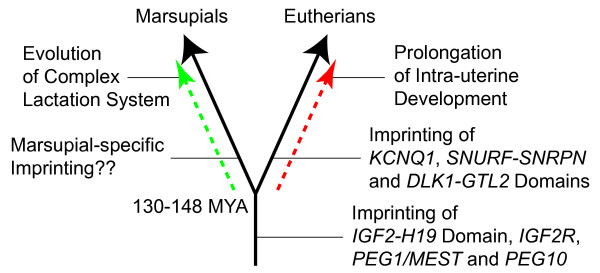
**Summary illustration**. The branched black arrow represents the evolutionally divergence between marsupials and eutherians which occurred at least 130-148 million years ago. The broken red arrow represents the evolution of eutherian-type gestation including the prolongation of inter-uterine development with a chorioallantoic placenta. The broken green arrow represents the evolution of the advanced complex lactation system as one of the remarkable and specialised features of marsupials. The acquisition of genomic imprinting in the *KCNQ1 *domain, accompanied with gene dosage reduction of *CDKN1C *and *PHLDA2*, occurred only in the evolution of the eutherian linage as well as the *SNURF-SNRPN *and *DLK1-GTL2 *domains [29,32]. On the other hand, imprinting of *IGF2-H19 *domain, *IGF2R, PEG1/MEST *and *PEG10 *occurred before the divergence of marsupials [26-28,30,31,33]. This study and others provide evidence that imprinting occurred at two critical time points during the evolution of mammals. For the third time point, whether marsupial-specific imprinting occurred or not, is currently still unknown.

The high level of mRNA expression in the trilaminar yolk sac placenta and the protein localisation to the cytoplasm of trophoblast cells suggest that tammar PHLDA2 is functional in their placenta. The lack of imprinting in the tammar *PHLDA2 *confirms an earlier conclusion that acquisition of genomic imprinting to the *KCNQ1 *domain occurred specifically in the eutherian lineage after the divergence of therian mammals into marsupials and eutherians, despite the fact that imprinting of the adjacent *IGF2-H19 *domain arose before the marsupial-eutherian split (Figure 5). Thus genomic imprinting of the *KCNQ1 *domain might have contributed to the development of complex placentation and the lengthening of gestation in the eutherian lineage by reducing gene dosage of negative regulators for both embryonic and placental growth.

## Methods

### Animals and tissue collection

Tammar wallabies of Kangaroo Island origin were maintained in our breeding colony in grassy, outdoor enclosures. Lucerne cubes, grass and water were provided *ad libitum *and supplemented with fresh vegetables. Fetuses and yolk sac placenta tissue were collected between days 21 and 26 of the 26.5 days gestation as previously described [24,42]. Experimental procedures conformed to Australian National Health and Medical Research Council (2004) guidelines and were approved by the Animal Experimentation Ethics Committees of the University of Melbourne.

### Amplification of tammar *PHLDA2 *sequence

The following primer pair for the amplification of the 272 bp tammar *PHLDA2 *fragment was designed from the highly conserved region in the multi-species sequence alignment:

272 forward 5'-GCGAGGGCGAGCTGGAGAAGCG-3'

272 reverse 5'-GATGGCCGCGTTCCAGCAGCTCT-3'

Thirty five cycles of PCR amplification were carried out in 25 μl total volume with 5-10 ng tammar cDNA from the yolk sac placenta using 0.5 U "*TaKaRa Ex Taq *Hot Start Version" (TaKaRa), 10 pmol each primers and 5 nmol each dNTP mixture under the following cycle conditions: 96°C × 15 s, 60°C × 30 s and 72°C × 30 s. PCR product was purified by "ExoSAP-IT" (GE) before sequencing. The 3' terminal of *PHLDA2 *mRNA was determined by "3' RACE System for Rapid Amplification of cDNA Ends" (Invitrogen) using the same forward primer described above as the gene specific primer. The intronic sequence was amplified by genomic PCR under the same conditions described above with 25 ng genomic DNA and the following primer pair:

Exon1 forward 5'-CGACTTCCGCTGCCCCGACG-3'

Exon2 reverse 5'-AAGACAAGGTCCCCATCGAG-3'

### Calculation of the amino acid sequence homology

The percentage homology of the amino acid sequence in the PH domain between tammar and multiple species was calculated using the homology search program in the "GENETYX-MAC" software.

### QPCR

Quantification of tammar *PHLDA2 *mRNA was performed using "Mx3000P QPCR System" (Agilent Technologies) with "Brilliant II SYBR Green QPCR Master Mix" (Agilent Technologies) under the following cycle conditions: 95°C × 15 s, 60°C × 30 s, 72°C × 30 s. The following primer pairs were used for the amplifications of *PHLDA2 *and *ACTB *as control:

*PHLDA2 *forward 5'-AGCCTCTTTCAGCTGTGGAA-3'

*PHLDA2 *reverse 5'-AAAATAGACGTGTTTGGCCG-3'

*ACTB *forward 5'-TTGCTGACAGGATGCAGAAG-3'

*ACTB *reverse 5'-AAAGCCATGCCAATCTCATC-3'

Total RNA was extracted from the fresh frozen fetal tissues and yolk sac placentas using "TRI Reagent Solution" (Applied Biosystems) and reverse transcribed using "SuperScript III First-Strand Synthesis System" (Invitrogen) with Oligo(dT) primer.

### Immunohistochemistry

Tissue sections (8 μm) were treated with 5% hydrogen peroxide in dH_2_O for 15 min to quench endogenous peroxidase activity. Slides were blocked in 10% normal goat serum in 0.1% BSA/TBS. Mouse monoclonal antibody raised against a partial recombinant human PHLDA2 (ABNOVA, H00007262-M01) was applied to sections at a 1:100 dilution at 4°C overnight. Antibody binding was detected with goat anti-mouse biotinylated secondary antibody (Dako) and amplified using the "Strept ABC Complex/HRP" (Dako). Antibody localisation was visualised using "Liquid DAB+ Substrate-Chromogen System" (Dako). Tissues were counterstained with haematoxylin.

### Allelic expression analysis

RNA was isolated using the "ISOGEN" (Nippongene). Extracted RNA was then treated with DNase (RT grade; Nippongene) at room temperature for 1 hr. Reverse transcription was performed using "SuperScript III First-Strand Synthesis System" (Invitrogen) with Oligo(dT) primer. RT-PCR amplifications were carried out at the same conditions as described in the previous section for the fetus #1 and #2, and 30 cycles with 68°C of annealing temperature for the fetus #3 and the pouch young #1, using following primer pair:

Exon1 forward 5'-CGACTTCCGCTGCCCCGACG-3'

Intron reverse 5'-TAGAGACTCCAGGAGCTGGC-3'

Three percent agarose gels were used for the electrophoresis. For amplification of *IGF2*, PCR conditions were the same in the previous section except the annealing temperature was 65°C and the primer pair:

Intron forward 5'-GACTCCACTTTCTTCCTTCCCTT-3'

Exon reverse 5'-AAAGCATGGCAGCCCACACT-3'

PCR products were purified by "ExoSAP-IT" (GE) before sequencing.

## Authors' contributions

SS conceived and designed the research, carried out all the analyses and drafted the manuscript. MBR and GS collected the embryos and placentas. GS, TK-I, FI and MBR participated in the design and coordination of the study and edited the manuscript. All authors read and approved the final manuscript.
